# Expression of estrogen receptor and progesterone receptor in endometrial cancer and their correlations with clinicopathological features

**DOI:** 10.3389/fonc.2026.1939245

**Published:** 2026-10-06

**Authors:** En Ping Li, Yu Hong, Jie Zhen Wei, Chun Ke Li, Hai Qi Liang

**Affiliations:** 1 Department of Obstetrics and Gynecology, Kiang Wu Hospital, Macau SAR, China; 2 Department of Pathology, Kiang Wu Hospital, Macau SAR, China

**Keywords:** Allred score, clinicopathological characteristics, endometrial carcinoma, estrogen receptor, progesterone receptor

## Abstract

**Objective:**

To investigate the expression levels of estrogen receptor (ER) and progesterone receptor (PR) in endometrial carcinoma (EC) and their correlations with standardized clinicopathological parameters in a single-center surgical cohort.

**Methods:**

Retrospective clinical and pathological data of 78 EC patients receiving primary surgery at the Department of Obstetrics and Gynecology of Kiang Wu Hospital between Jan 2016 and Aug 2022 were collected. ER/PR expression was quantified via immunohistochemistry (IHC) using the Allred scoring system. Dichotomous receptor positivity (Allred 0–2 negative/3–8 positive) and continuous total Allred scores were analyzed separately. Fisher’s exact test or χ² test was used for categorical comparison; Kruskal-Wallis H test with adjusted *post-hoc* pairwise tests and Wilcoxon rank-sum test were applied for ordinal Allred score data. Exact P values, test statistics and degrees of freedom were reported for all analyses.

**Results:**

The overall cohort included 71 endometrioid carcinoma (EC) and 7 non-endometrioid carcinoma (NEC) cases. ER and PR positive rates were significantly higher in endometrioid lesions (97.2% and 94.4%) than non-endometrioid subtypes (57.1% for both, P = 0.018/P = 0.018). Continuous Allred scores of ER and PR declined significantly with worsening tumor differentiation (all adjusted P<0.05). For FIGO staging, significantly reduced ER and PR Allred scores were observed in Stage II compared with Stage I. The single Stage-III case was reported descriptively and was not included in formal statistical testing. Patients with lymph node metastasis exhibited lower PR Allred scores (P = 0.021), while ER expression showed no statistically significant difference (P = 0.076). No significant associations were found between ER/PR scores and depth of myometrial invasion or lymphovascular space invasion (LVSI) (all P>0.05).

**Conclusion:**

Reduced ER and PR expression correlates with poorer tumor differentiation and advanced FIGO stage in endometrioid endometrial carcinoma; PR loss is independently associated with lymph node metastasis. This study only identifies crude cross-sectional correlations without survival or multivariable adjustment data, so ER/PR may merely serve as auxiliary morphological markers reflecting tumor aggressive potential, rather than standalone indicators for prognosis or individualized hormone therapy guidance.

## 1 Introduction

Endometrium acts as a hormone-sensitive target organ regulated by estrogen and progesterone via nuclear estrogen receptor (ER) and progesterone receptor (PR), driving regular proliferative and secretory cyclic changes. Long-term unopposed estrogen exposure without progesterone antagonism (obesity, PCOS, chronic anovulation) promotes endometrial hyperplasia and malignant transformation into endometrial carcinoma (EC) (1). Aberrant ER/PR expression is tightly linked to EC initiation, progression, and hormone treatment responsiveness (2–5).

Immunohistochemical analysis revealed that ER and PR are widely expressed in various subtypes of endometrial cancer. However, receptor expression levels exhibited significant heterogeneity, closely related to tumor biology. The loss of estrogen receptor/progesterone receptor (ER/PR) in cancer cells was significantly associated with aggressive clinicopathological features such as high histological grade (G3), deep myometrial invasion, lymphovascular space invasion (LVSI), and lymph node metastasis (6–9). Furthermore, the loss of ER/PR in both tumor cells and the surrounding myometrium was independently associated with disease progression and poor prognosis (6).

The prognostic and predictive value of ER/PR status is increasingly being validated. Patients with ER/PR-positive tumors have significantly higher disease-free survival (DFS) compared to receptor-negative patients (10); tumors with PR immunohistochemical expression >50% have a higher response rate to progestin therapy (11, 12), while PR negativity predicts resistance to hormone therapy, necessitating alternative treatments (13, 14). Newly emerging evidence also integrates ER/PR into risk stratification assessments based on molecular subtypes. A three-tiered ER/PR expression scoring system enhances prognostic differentiation, particularly in the highly heterogeneous “no specific molecular signature” (NSMP) molecular subgroup (15, 16). Furthermore, combined assessment of ER/PR with CA-125, lymphovascular invasion (LVSI), and histological grade improves the predictive accuracy of lymph node metastasis and recurrence risk.

However, multiple unresolved gaps remain in current clinical practice:

1. No universal consensus on standardized IHC Allred cut-off thresholds for EC ER/PR positivity across ethnic populations (5, 17);
2. Most single-center cohorts lack stratified analysis of continuous Allred score gradients versus dichotomous positive/negative status;
3. Few local Macau Chinese cohorts have reported ER/PR expression distribution across full FIGO stages and all pathological risk factors;
4. Existing literature rarely distinguishes ER isoforms (ERα/ERβ) and PR isoforms (PR-A/PR-B) when interpreting receptor downregulation mechanisms;
5. Most retrospective studies fail to separate two analytical strategies (binary positivity vs continuous Allred score) and their respective clinical meanings.

Key limitation of the present study to declare in advance: Formal molecular testing (POLE, MMR, p53) was not routinely performed in our hospital during 2016–2022, so correlation between ER/PR and molecular subtypes cannot be analyzed. In addition, we only collected perioperative pathological data without long-term survival follow-up or postoperative hormone therapy response records, thus cannot evaluate predictive or prognostic performance of ER/PR.

This single-center retrospective study recruited a consecutive surgical cohort of Chinese EC patients in Macau. We separately analyzed dichotomous ER/PR positivity rates and continuous Allred expression scores, systematically exploring their correlations with histological subtype, differentiation grade, myometrial invasion, LVSI, lymph node metastasis and FIGO stage. We aim to supplement regional real-world pathological data and provide reference for local pathological risk evaluation of EC.

## 2 Materials and methods

### 2.1 Study subjects

Clinical data of EC patients receiving surgical treatment at the Department of Obstetrics and Gynecology of Kiang Wu Hospital between January 2016 and August 2022 were retrospectively enrolled. Inclusion criteria were as follows:

1. Preoperative EC diagnosis confirmed via hysteroscopy or diagnostic curettage pathology (including endometrioid carcinoma, clear cell carcinoma, serous carcinoma, carcinosarcoma, etc.);
2. Primary surgical resection without prior radiotherapy, chemotherapy, immunotherapy or other anti-tumor treatments;
3. Completion of standardized comprehensive surgical staging with full operative records;
4. Complete pathological reports documenting histological subtype, tumor differentiation grade, depth of myometrial invasion, lymphovascular space invasion (LVSI) and lymph node metastasis status;
5. Valid IHC testing results of ER and PR on endometrial tumor specimens.

This retrospective study was approved by the Institutional Review Board of Kiang Wu Hospital (Approval No.202403). All patients provided written informed consent for the use of their clinical and pathological data for research purposes.

### 2.2 Surgical procedures

All patients received standardized surgical-pathological staging procedures: peritoneal fluid or peritoneal lavage fluid was collected for cytological examination; intraperitoneal organs were comprehensively explored with biopsy of suspicious lesions; total hysterectomy plus bilateral salpingo-oophorectomy was performed, combined with pelvic and/or para-aortic lymphadenectomy at the surgeon’s discretion.

### 2.3 Histopathological examination and immunohistochemistry

All postoperative specimens were fixed in 10% formalin, embedded in paraffin, and routinely stained with hematoxylin-eosin (HE). Histopathological diagnosis of endometrial lesions was performed in accordance with the 2014 WHO Classification of Female Genital Tumors (18). All cases were uniformly restaged according to the 2009 FIGO Endometrial Cancer Staging System to eliminate staging heterogeneity across the 2016–2022 enrollment period.

Immunohistochemical staining was conducted using the streptavidin-peroxidase (S-P) method on automated Ventana platform. Ready-to-use monoclonal primary antibodies against ER (clone SP1) and PR (clone 1E2) manufactured by VENTANA (USA) were applied following the manufacturer’s protocols.

#### 2.3.1 IHC scoring criteria

The Allred scoring system was adopted to evaluate ER and PR expression, combining the proportion of positive tumor cells (score range: 0-5) and staining intensity (score range: 0-3). The total Allred score ranged from 0 to 8 (as shown in **Table 1**):

- Total score 0-2: Negative ER/PR expression (binary negative group)
- Total score 3-8: Positive ER/PR expression (binary positive group) Two analytical dimensions were applied separately:

1. Dichotomous analysis: Positive vs negative rate (suitable for comparing overall receptor prevalence across subtypes);
2. Continuous ordinal analysis: Total Allred score gradient (reflect gradual downregulation of receptor expression with tumor progression).

**Table 1:** Allred Scoring System for ER and PR Immunostaining.

Score	Proportion of positive tumor cells	Staining intensity
0	0%	No Staining
1	<1%	Weak
2	1-10%	Moderate
3	11-33%	Strong
4	34-66%	—
5	>66%	—

### 2.4 Statistical analysis

Statistical analyses were performed using SPSS 19.0 software.

1. Categorical variables (histological subtype, LVSI, nodal metastasis, binary ER/PR positive/negative):

- Group expected count ≥5: Pearson χ² test;
- Any cell expected count <5: Fisher’s exact test.

2. Ordinal continuous Allred scores failed normality test (Kolmogorov-Smirnov), presented as median (interquartile range, M(IQR)) + mean rank for nonparametric comparison:

- Two-group comparison: Wilcoxon rank-sum test;
- Three/multi-group comparison: Kruskal-Wallis H test, followed by Dunn-Bonferroni *post-hoc* test with adjusted P values for multiple pairwise comparisons to control type I error.

3. All statistical results report exact P values, test statistic and corresponding degrees of freedom; P < 0.05 (two-tailed) was defined as statistically significant.

## 3 Results

A total of 78 EC patients were included in this study, consisting of 71 cases of endometrioid carcinoma and 7 cases of non-endometrioid carcinoma (clear cell carcinoma, serous carcinoma and carcinosarcoma). All baseline information was listed in **Table 2**. Representative HE and IHC staining images of pathological samples are shown in **Figure 1**.

**Table 2:** Baseline clinicopathological characteristics of all 78 EC patients.

Parameter	Case number (%)
Total patients	78 (100)
Median age (IQR), years	56 (49-63)
Menopausal status	
Premenopausal	29 (37.2)
Postmenopausal	49 (62.8)
Histological subtype	
Endometrioid carcinoma	71 (91.0)
Non-endometroid carcinoma (serous/clear cell/carsinosarcoma)	7 (9.0)
Differentiation grade (endometrioid only)	
G1 well-differentiated	33 (46.5)
G2 moderately differentiated	25 (35.2)
G3 poorly differentiated	13 (18.3)
FIGO 2009 Stage	
I	62 (79.5)
II	8 (10.3)
III	1 (1.3)
Myometrial invasion	
No invasion	6 (7.7)
<1/2 myometrium	43 (55.1)
≥1/2 myometrium	22 (28.2)
LVSI status	
Negative	54 (69.2)
Positive	17 (21.8)
Lymph node assessment	
Lymphadenectomy performed	66 (84.6)
No lymph node sampling	12 (15.4)
Lymph node metastasis (only n=66 assessed)	
Negative	56 (84.8)
Positive	10 (15.2)

**Figure 1:**
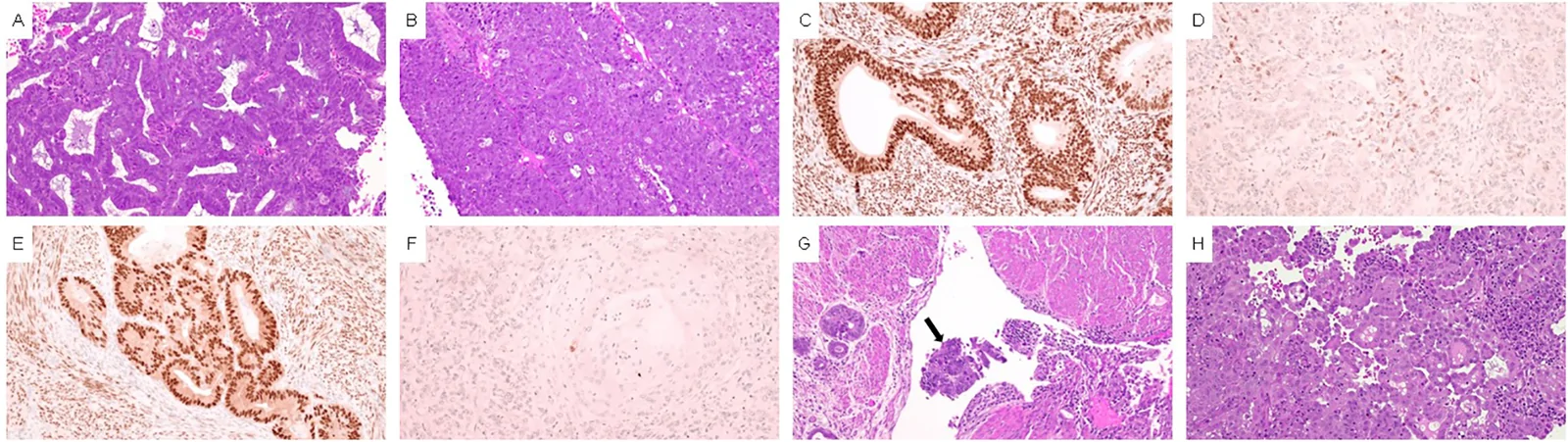
Representative HE and IHC staining images. **(A)** G1 endometrioid carcinoma HE; **(B)** G3 poorly differentiated endomtrioid carcinoma HE; **(C)** Strong ER IHC (Allred score 8); **(D)** Weak ER IHC (Allred score 3); **(E)** Strong PR IHC (Allred score 8); **(F)** PR negative staining (Allred score 2); **(G)** LVSI positive HE image; **(H)** Non-endometrioid serous carcinoma HE. All images captured at 200x magnification, scale bar = 100μm.

### 3.1 ER/PR binary positive rates by histological subtype

The positive rates of ER and PR were 97.2% and 94.4% in endometrioid carcinoma, both significantly higher than those in non-endometrioid carcinoma (P < 0.05, **Table 3**).

**Table 3:** Binary ER/PR positive rates: endometrioid vs. non-endometrioid carcinoma.

ER/PR status	Endometrioid (n=71) [n(%)]	Non-endometrioid (n=7) [n(%)]	P value (Fisher exact)
ER positive	69(97.2)	4(57.1)^*^	0.018
PR positive	67(94.4)	4(57.1)^*^	0.018

### 3.2 Allred score by tumor differentiation (Endometrioid Only, n=71)

Total Allred scores of ER and PR expression declined significantly with increasing tumor differentiation grade (worse differentiation), with statistically significant differences among groups (P < 0.05, **Table 4**).

**Table 4:** ER/PR Allred score (median (IQR), mean rank) across differentiation grades.

Grade	ER M(IQR), mean rank	PR M(IQR), mean rank
G1 (n=33)	7 (6-8), 42.8	7 (6-8), 46.7
G2 (n=25)	5 (3-7), 31.1	4 (2-6), 27.9
G3 (n=13)	4 (0-6), 25.7	3 (0-5), 22.5

Kruskal-Wallis H for ER = 16.24, df=2, adjusted P<0.001; vs G1: G2 P = 0.012, G3 P<0.001.

Kruskal-Wallis H for PR = 23.71, df=2, adjusted P<0.001; vs G1: G2 P<0.001, G3 P<0.001.

### 3.3 Myometrial invasion & LVSI Allred scores

No significant correlation was observed between ER/PR expression and the depth of myometrial invasion (P > 0.05). Patients with LVSI exhibited lower ER and PR expression than LVSI-negative patients, but the intergroup difference was not statistically significant (P > 0.05, **Table 5**).

**Table 5:** ER/PR Allred score stratified by myometrial invasion and LVSI.

	ER M(IQR), mean rank	PR M(IQR), mean rank
Myosmetrial invasion^a^		
None (n=6)	6 (4-8), 35.7	6 (4-8), 36.1
<1/2 (n=43)	6 (3-7), 34.6	6 (3-7), 36.8
≥1/2 (n=22)	6 (3-8), 38.8	6 (3-7), 34.4
Lymphovascular space invasion(LVSI)^b^		
Positive (n=17)	5 (3-7), 25.6	5 (3-7), 27.7
Negative (n=54)	6 (3-8), 33.4	6 (3-8), 32.7

^a^ Kruskal-Wallis ER H = 0.42, df=2, P = 0.811; PR H = 0.19, df=2, P = 0.908.

^b^ Wilcoxon ER Z=-1.53, P = 0.125; PR Z=-1.14, P = 0.253.

### 3.4 Lymph node metastasis

Lymph-node-metastasis-related analyses were restricted to the 66 patients who underwent lymph-node assessment; 12 patients without lymph-node sampling were excluded from this part of analysis. Patients with lymph node metastasis showed lower ER and PR expression than those without lymph node metastasis. Only PR expression presented a statistically significant difference between the two groups (P < 0.05, **Table 6**).

**Table 6:** ER/PR Allred score stratified by nodal status (only n=66 evaluable; 12 patients without lymph-node sampling excluded).

Node status	ER M(IQR), mean rank	PR M(IQR), mean rank
Node negative (n=56)	6 (3-8), 30.7	6 (3-8), 31.3
Node positive (n=10)	5 (3-7), 20.9	4 (3-6), 16.4^c^

^c^ :Wilcoxon ER Z=-1.78, P = 0.076; PR Z=-2.31, P = 0.021.

### 3.5 FIGO stage Allred score

Formal non-parametric statistical comparison was performed only between FIGO Stage I (n=62) and Stage II (n=8). The Stage-III subgroup contained only a single patient and was reported descriptively and was not included in formal hypothesis testing.

As shown in **Table 7**, comparison between Stage I and Stage II showed significantly reduced ER and PR Allred scores in Stage-II disease (ER: Wilcoxon rank-sum Z =-2.71, adjusted P = 0.007; PR: Z =-3.04, adjusted P = 0.002). The single Stage-III case had an ER Allred score of 5 and PR Allred score of 6; no statistical inference can be drawn from this isolated observation.

**Table 7:** ER/PR Allred score across FIGO 2009 stages.

Clinical stage	ER M(IQR), mean rank	PR M(IQR), mean rank
Stage I (n=62)	6 (3-8), 37.4	6 (3-8), 38.4
Stage II (n=8)	5 (3-7), 27.3	5 (3-7), 27.5
Stage III (n=1)^d^	5 (5-5),-	6 (6-6), -

^d^ Descriptive presentation only; not included in formal statistical testing.

## 4 Discussion

The incidence of EC is rising globally with a trend toward younger onset. In multiple developed regions, EC has surpassed cervical cancer as the most prevalent malignant tumor of the female reproductive tract. Widespread cervical cancer screening and HPV vaccination have effectively reduced cervical cancer morbidity, providing novel research directions for EC with similar biological behaviors (19). At present, EC can be diagnosed via ultrasonography, diagnostic curettage or hysteroscopy combined with pathological examination; however, these invasive procedures are costly and associated with complications, limiting their application for mass early screening. With advances in molecular pathology, tissue and serum tumor biomarkers have become core indicators for EC diagnosis, treatment stratification and survival prognosis evaluation.

ER and PR are widely expressed in normal endometrium, endometrial hyperplasia and EC, serving as critical prognostic biomarkers for endometrial lesions. As steroid nuclear receptors, ER and PR mediate estrogen and progesterone bioactivity in endometrial tissue: estrogen promotes endometrial proliferation, while progesterone counteracts estrogen-induced proliferative activity. Persistent unopposed estrogen stimulation without progesterone protection triggers endometrial hyperplasia and subsequent malignant transformation (17). EC is histologically divided into two major subtypes: type I estrogen-dependent endometrioid carcinoma (accounting for over 80% of all EC cases) with high ER/PR positivity and favorable prognosis, and type II non-estrogen-dependent carcinoma (including serous and clear cell carcinoma) with frequent ER/PR loss and poor clinical outcomes (20, 21). Consistent with domestic epidemiological data, our Macau cohort included 91% endometrioid carcinoma and 9% non-endometrioid carcinoma. Hormone receptor status is a core pathological marker to distinguish Type I/II subtypes (21). Our data confirmed ER/PR positive rates are far higher in endometrioid lesions (97.2%/94.4%) versus rare non-endometrioid subtypes (57.1%/57.1%) (*P* = 0.018), consistent with global cohort findings (4, 21).

Two main nuclear ER isoforms exist: ERα mediates proliferative estrogen signaling, while ERβ exerts anti-proliferative effects; PR has PR-A and PR-B isoforms, with PR-B as the dominant progesterone effector. Malignant progression disrupts the balance of ER/PR isoform expression: poorly differentiated tumor cells downregulate total ER/PR transcription and translation, reducing Allred score gradients stepwise from G1 to G3, which matches our ordinal score results. Most prior literature only reported binary positive/negative rates; our study additionally supplies continuous Allred median and interquartile ranges, providing quantitative gradient data unique to this local Chinese cohort.

Postoperative adjuvant therapy for EC is determined based on traditional high-risk clinicopathological factors, including deep myometrial invasion, poor tumor differentiation (G3), non-endometrioid histological subtypes, LVSI and lymph node metastasis (22). Most existing prognostic prediction models for EC survival are built upon these conventional pathological parameters. In this study, ER and PR Allred scores declined stepwise with poorer tumor differentiation, indicating that malignant transformation impairs receptor synthesis, and advanced malignant phenotypes may completely abolish ER/PR expression capacity (4). For FIGO staging, statistically significant reduction of ER/PR Allred scores was observed between Stage I and Stage II. Only one patient was classified as FIGO Stage III; this case is presented descriptively and cannot support any formal statistical comparison. Among patients receiving lymphadenectomy, only PR expression showed statistically significant decline in nodal metastasis cases (P = 0.021), implying PR loss may better reflect metastatic potential than ER downregulation in endometrioid EC. No statistical correlation was found between receptor levels and myometrial invasion depth or LVSI, possibly due to moderate sample size and uneven subgroup distribution, consistent with some regional Chinese retrospective reports (23, 24), which requires further validation with expanded patient cohorts.

Though the general trend of ER/PR loss in high-risk EC has been repeatedly reported in Western and inland Chinese cohorts, this study has several original supplementary points:

1. First real-world Macau Chinese surgical cohort reporting full Allred continuous score distributions rather than only dichotomous positive rates;
2. Strict separation of two independent analytical modes (binary positivity vs ordinal Allred gradient) with clear statistical specification;
3. Stratified exclusion of patients without lymph node sampling when analyzing nodal metastasis correlation, avoiding data mixing bias;
4. Detailed subgroup comparison of late FIGO Stage II limited cases, providing reference for small-volume intermediate-stage EC.

This study lacks two important dimensions: treatment response correlation and isoform-specific IHC testing. We clarify that hormone therapy follow-up data and ERα/ERβ, PR-A/PR-B isoform antibodies were unavailable during the study period, which is listed clearly in limitations; we propose future prospective research to detect receptor isoforms and track postoperative hormone treatment response.

### Critical study limitations

1. Single-center retrospective design with limited total sample size (n=78); non-endometrioid (n=7) and FIGO III (n=1) subgroups are extremely imbalanced, causing selection bias;
2. No standardized molecular subtype testing (POLE, MMR, p53) was performed for all specimens, so ER/PR-molecular subtype correlations cannot be analyzed;
3. Only cross-sectional perioperative pathological data collected, without long-term disease-free or overall survival follow-up; cannot validate prognostic value of ER/PR;
4. No multivariable logistic/Cox regression to adjust confounding factors (age, menopause, grade, stage) when evaluating ER/PR correlation with nodal metastasis;
5. No isoform-specific IHC (ERα/ERβ, PR-A/PR-B) or postoperative hormone therapy response records to assess predictive capacity for progestin treatment;
6. 12 patients did not receive lymph node sampling and were excluded from nodal metastasis subgroup analysis, reducing statistical power for this comparison;
7. Only surgical EC patients were enrolled, excluding biopsy-only unresectable advanced cases, limiting population representativeness.

## 5 Conclusion

In this Macau Chinese single-center surgical cohort, gradual downregulation of ER and PR continuous Allred expression scores correlates with worsening tumor differentiation and FIGO Stage II progression in endometrioid endometrial carcinoma; isolated PR reduction is significantly associated with lymph node metastasis. Binary ER/PR positivity rates are markedly lower in rare non-endometrioid subtypes. Given the study’s inherent limitations including cross-sectional design, absent survival data, lack of multivariable adjustment and molecular classification, ER/PR can only be regarded as auxiliary pathological markers reflecting tumor aggressive morphology, and cannot be independently used for prognosis judgment or individualized hormone therapy selection in clinical practice.

## Data Availability

The datasets presented in this article are not readily available because the raw clinical and pathological datasets supporting this study cannot be publicly released due to hospital privacy-management regulations, but de-identified aggregated analysis data can be provided to qualified researchers upon reasonable formal request to corresponding author Yu Hong. Requests to access the datasets should be directed to Yu Hong. xmdehy@126.com.
